# Exponential spectro-temporal modulation generation

**DOI:** 10.1121/10.0003604

**Published:** 2021-03-02

**Authors:** Trevor A. Stavropoulos, Sittiprapa Isarangura, Eric C. Hoover, David A. Eddins, Aaron R. Seitz, Frederick J. Gallun

**Affiliations:** 1 Brain Game Center for Mental Fitness and Well-being, University of California, Riverside, California 92521, USA; 2 Department of Communication Sciences and Disorders, Mahidol University, Bangkok, Thailand; 3 Department of Hearing and Speech Sciences, University of Maryland, College Park, Maryland 20742, USA; 4 Auditory and Speech Science Laboratory, University of South Florida, Tampa, Florida 33612, USA; 5 National Center for Rehabilitative Auditory Research, Portland VA Medical Center, Portland, Oregon 97239, USA; 6 Otolaryngology/Head and Neck Surgery, Oregon Health and Science University, Portland, Oregon 97239, USA

## Abstract

Traditionally, real-time generation of spectro-temporally modulated noise has been performed on a linear amplitude scale, partially due to computational constraints. Experiments often require modulation that is sinusoidal on a logarithmic amplitude scale as a result of the many perceptual and physiological measures which scale linearly with exponential changes in the signal magnitude. A method is presented for computing exponential spectro-temporal modulation, showing that it can be expressed analytically as a sum over linearly offset sidebands with component amplitudes equal to the values of the *modified Bessel function of the first kind*. This approach greatly improves the efficiency and precision of stimulus generation over current methods, facilitating real-time generation for a broad range of carrier and envelope signals.

## I. INTRODUCTION

Spectro-temporal modulation (STM) is of great interest in psychoacoustics and auditory physiology because of its relevance to speech decoding^1–3^ as well as the broad applicability of the modulation-based linear-systems approach to parametric investigations. Investigations involving STM have used a variety of modulator shapes and carrier types. The two most common carriers are broadband noise and tonal complexes, although the details of each vary from study to study.

Creating STM with modulation that is sinusoidal on a logarithmic amplitude scale through explicit evaluation of the time-domain representation is computationally costly—far too costly to be practical for generating stimuli for presentation during an experiment. To make use of such exponential STM, stimuli frequently must be calculated in advance using fixed parameters, which limits the psychophysical methods that can be used. Additionally, an insufficiently robust set of pre-generated stimuli requires limiting adaptive procedures or risks design flaws due to the recognizability of frozen noise.^4^ As a result of these computational constraints and the relative simplicity of its form, many studies use STM with a modulation envelope that is sinusoidal on a linear amplitude scale. A stimulus comprised of the sum of *N* simple carrier tones decomposes into 3*N* different tones under linear modulation. However, one of the oldest, best-established psychoacoustics principles is that sensitivity to sound intensity is *logarithmic* not *linear.*^5^ This is a critical assumption of most estimates of the internal excitation in response to an acoustic stimulus.^6–9^ Thus, implementing the desired modulation pattern on a logarithmic amplitude scale ensures a similar pattern to a first approximation at the level of excitation, which translates to applying exponential rather than linear modulation.

Existing methods for achieving exponential STM tend to be either unsuitable for real-time generation because they are too computationally intensive or poorly suited for research due to constraints or inherent imprecision. Further, some attempts to limit the cost of generating stimuli, such as making use of low carrier-tone densities, have led to unwanted stimulus artifacts.^10^ A computationally efficient method for generating exponential STM from independent carrier tones in the frequency domain is presented here along with metrics comparing the resultant stimulus with the explicit form and an existing alternative.

## II. STM

When generating signals that vary in intensity as a function of time and frequency, it is necessary to consider how variation is represented by the amplitude and phase as a function of frequency. An inherent feature of any finite, practical auditory filter (like that of the human auditory system) is some degree of frequency selectivity. Given any such auditory filter and using noise composed of stationary, uncorrelated tones, a tone-density sensitivity threshold exists above which the system is insensitive to further increases in tone density.^11^ This is part of the basis for the common practice of generating noise in the frequency domain as a sum of a limited number of stationary, uncorrelated tones distributed through a frequency band.

The fact that complex stimuli can be represented as a linear sum of simple carrier tones has long been appreciated (and exploited). It allows for the leveraging of powerful mathematical technology, like the discrete Fourier transform (DFT), to generate stimuli much more rapidly than could otherwise be done. Improvements to the computational efficiency of generating stimuli allows for the flexible generation of custom stimuli in real-time, circumventing the aforementioned issues that arise when relying on pre-generated stimuli. Computational complexity and, thus, generation time can be limiting factors for real-time generation of robust, broad-spectrum noise.

A generalized form that can be used to represent the time waveform, *S*(*t*), of STM generated from noise that is composed as of a sum of *N* pure sine wave carrier tones is given by

$$S(t)=\sum_{n=1}^{N}\overset{\mathrm{Carrier}\;\;\mathrm{tone}}{\overset{︷}{A_{n}\;\text{sin}\;(2\;\pi f_{n}t+\phi_{n})}}\overset{\mathrm{Modulator}}{\overset{︷}{M(f_{n},t)}},$$ (1)

where each carrier tone is described by an amplitude *A_n_*, a frequency *f_n_*, and a phase $\phi_{n}$, and $M(f_{n},t)$ is the modulator—a function of both time and carrier frequency. This representation cleanly separates the properties of the underlying noise, such as the different statistical distributions and spectral shaping, which would be reflected in the carrier tone amplitudes, *A_n_*, from the modulator function, $M(f_{n},t)$. With different choices for the modulator, this form can represent both exponential and linear STM, as well as their respective spectral modulation (SM) and temporal modulation (TM) counterparts. Starting with this general, conceptual model helps establish a common framework in which linear and exponential modulations can be developed and compared without unnecessarily constraining how they are applied.

### A. Linear modulation

Linear modulation is often used either because of the simplicity of its form or the efficiency of generating such a stimulus in the frequency domain. Using the above generalized form, simple linear modulation could be expressed as

$$M_{\text{Lin}}(f,t)=1+m\;\;\text{sin}\;(2\;\pi \;\omega \;t+\Phi (f)),$$ (2)

where *m* is the linear modulation depth and a value in the range [0,1),^12^ *ω* is the TM rate in Hz, and Φ(f) is a function that determines the modulation phase. Application of this modulator to the carrier-tone sum results in each tone receiving a sinusoidal temporal envelope with an envelope phase shift determined by its frequency, which is responsible for creating the progressive spectral offset that is characteristic of STM. A scaling prefactor for normalization has been dropped for convenience and clarity. Direct use of this form of the modulator would change the root-mean-square (RMS) level of the carrier, requiring either the level of the output stimulus to be rescaled or the normalization constant to be calculated beforehand. In the literature, the linear modulation depth is frequently reported as $20\;\;{\text{log}}_{10}m$.

Typically, SM that is periodic on a logarithmic frequency (octave) scale is desired, in which case, the envelope phase would be

$$\Phi (f)=2\;\pi \;\Omega \;\;{\text{log}}_{2}\left(f/f_{0}\right)+\Phi_{0},$$ (3)

where Ω is the spectral density of the modulation in terms of cycles per octave, $\Phi_{0}$ is the phase shift of the entire modulation envelope, and *f*_0_ is any reference frequency (typically the lower-bound of the spectral domain of the noise, although in principle any value can be used as it only determines which frequency receives the modulator phase shift of $\Phi_{0}$). While Eq. (3) was included for clarity and completeness, it is not necessary to specify the spectral properties of the modulation envelope to derive a result for either linear or exponential STM and as such, the results apply broadly to any spectral relationship whether logarithmic, linear, or constant as is the case with TM. For notational convenience, the envelope phase of the *n*th carrier tone will be defined as $\Phi_{n}\equiv \Phi \left(f_{n}\right)$, although the results can be reinterpreted for continuous frequency distributions simply by reversing this substitution.

When $M_{\text{Lin}}$ [Eq. (2)] is substituted into the general form [Eq. (1)] as the modulator function *M* and the trigonometric product rules are applied, the product of this modulator and each carrier tone simplifies to the sum of three simple sine waves, representing a base carrier tone of frequency *f* and two sidebands of frequencies f+ω and f−ω [see Eq. (18) and Table I].

**TABLE I.** The transformation to apply to each carrier tone in order to apply linear modulation. Each carrier tone is replaced with a base tone and two sidebands of linearly offset frequency.

Linear modulation sidebands			
	Frequency	Amplitude^a^	Phase
Base	*f_n_*	*A_n_*	$\phi_{n}$
Upper band	$f_{n}+\omega$	$-\frac{1}{2}mA_{n}$	$\phi_{n}-\frac{\pi }{2}+\Phi_{n}$
Lower band	$f_{n}-\omega$	$\frac{1}{2}mA_{n}$	$\phi_{n}-\frac{\pi }{2}+\Phi_{n}$

^a^ Whereas it is improper to express amplitude as a negative value, it does greatly simplify the comparison with the exponential sidebands in this format. To recover the proper amplitudes and phase shifts, remove the factor of −1 appearing in an amplitude term and add a phase shift of π.

### B. Exponential modulation

Exponential modulation requires a more complex representation,

M Exp (f,t)=10(m/20) sin (2 π ω t+Φ(f)), (4)

where *m* is the modulation depth in decibels, a positive value representing the level difference between a peak or a valley and the midpoint, *ω* is the TM rate in Hz, and Φ(f) is a function that determines the modulation phase. Note that when *m* = 0 dB, the modulator is strictly equal to one, leaving the carrier tone unmodified. A significant downside to this form, and likely the reason it has been neglected in favor of the simpler linear modulator, is that the modulator can no longer be represented by sidebands determined by a simple trigonometric relationship as in the linear case.

Much like linear STM, exponential STM can be accurately represented as the sum over a limited number of sidebands [see Eq. (17) and Table II]. The full derivation of the sideband relationship can be found in Sec. III. Whereas the analytic solution contains a sum over an infinite number of sidebands, Sec. IV A discusses how the sum converges quickly enough that a very limited number of sidebands is sufficient to produce an accurate stimulus across the range of modulation depths required in practice.

**TABLE II.** The transformation to apply to each carrier tone in order to apply exponential modulation. Each carrier tone is replaced with a base tone and its associated sidebands. The first sidebands correspond to *k* = 1. The even-numbered sidebands use the appropriate *Even k* row, the odd-numbered sidebands use the respective *Odd k* row. The amplitude terms fall off exponentially for successive sidebands. For 20-dB peak-to-valley modulation (*m* = 10), by the fifth set of sidebands (*k* = 5), each subsequent amplitude term is more than 1 order of magnitude smaller than the prior.

Exponential modulation sidebands				
	Frequency	Band	Amplitude^a^,^b^,^c^	Phase
Base	*f_n_*	—	$I_{0}(M\prime )A_{n}$	$\phi_{n}$
Upper bands	$f_{n}+k\omega$	(Even *k*)	${\left(-1\right)}^{k/2}I_{k}(M\prime )A_{n}$	$\phi_{n}+k\Phi_{n}$
		(Odd *k*)	${\left(-1\right)}^{(k+1)/2}I_{k}(M\prime )A_{n}$	$\phi_{n}-\frac{\pi }{2}+k\Phi_{n}$
Lower bands	$f_{n}-k\omega$	(Even *k*)	${\left(-1\right)}^{k/2}I_{k}(M\prime )A_{n}$	$\phi_{n}-k\Phi_{n}$
		(Odd *k*)	${\left(-1\right)}^{(k-1)/2}I_{k}(M\prime )A_{n}$	$\phi_{n}-\frac{\pi }{2}-k\Phi_{n}$

^a^ The substitution M′≡(m/20) ln (10) is made for readability.

^b^ $I_{\nu }(z)$ is the modified Bessel function of the first kind of order *ν* and argument *z*.

^3^ See the footnote regarding negative amplitudes in Table I.

## III. DERIVATION

The goal is to manipulate the expression for the exponential modulator [Eq. (4)] so that it is expressed solely as a sum of pure tones with well-defined frequencies, amplitudes, and phases. Such a representation can then be exploited to *a proiri* calculate a STM stimulus in the frequency domain, reducing the entire computational cost of generating exponential STM to little more than that of a single DFT and potentially reducing the computational burden by several orders of magnitude.

### A. Overview

The first step is to find the Taylor expansion of the exponential to pull the sine wave in the exponent into a marginally more cooperative form, an infinite sum over powers of sine [Eq. (6)]. Next, the sine power reduction formula is used to rewrite each power of sine into a sum of sine and cosine terms [Eq. (10). The formula being invoked here is the result of the recursive application of the trigonometric product rules [such as 2  sin  θ  sin  ϕ=cos (θ−ϕ)−cos (θ+ϕ)]. However, because this formula has a different form for even and odd exponents of sine (as sine and cosine end up transforming back and forth with each additional power), these terms are temporarily split into a sum over the even terms, a sum over the odd terms, and the 0th term.

The result of this manipulation is a finite sum inside an infinite sum. The next step is to collect every trigonometric function with the same argument together and sum the amplitudes. To take the even sum, as an example, the first term of the outer sum (*m* = 2) is a scalar value multiplied by cos  2θ, and the second term of the outer sum (*m* = 4) is a cos  2θ term plus a cos  4θ term. Similarly, the third term of the outer sum (*m* = 6) is a sum of a cos  2θ term, a cos  4θ term, and a cos  6θ term. This expression becomes significantly simpler if the sums are rearranged and all of the cos  2θ terms are collected together, all of the cos  4θ terms are collected together, etc., effectively turning the nested sum inside out. The simplified representation that this yields is a sum over constant values [Eq. (11a)], cosine terms [Eq. (11b)], and sine terms [Eq. (11c)].

This new representation is in the form of a particularly well-studied function, the *modified Bessel function of the first kind*, $I_{\nu }(z)$ [Eq. (12)]. *Bessel functions*, of which $I_{\nu }(z)$ is included, appear throughout physics and engineering due to involvement in the representations of spherical harmonics. It is pertinent here to note that many mathematical packages include this function (besseli in matlab, for instance), and highly efficient numerical recipes exist for it as well (see Ref. 13). The expression collapses into a sum over sine and cosine terms with amplitudes equal to values of the *Bessel functions* [Eq. (15)].

Finally, the modulator expression is multiplied by its corresponding carrier tone [Eq. (1)], and the trigonometric product rules are applied to generate the final representation: a base tone plus sidebands that are linearly offset by integer multiples of the TM rate with amplitudes equal to values of the *modified Bessel function of the first kind* [Eq. (17)]. This form is closely parallel to the linear modulation form [Eq. (18)] albeit with a little more complexity. Nonetheless, the values of $I_{k}(z)$ drop off rapidly as *k* increases, especially in the range of physiologically relevant modulation (see the discussion on error in Sec. IV A for more details), allowing for high accuracy with the use of as few as ten sidebands.

### B. Complete derivation

The first step to finding a cleaner representation of the exponential modulation is to use the Taylor expansion of the exponential. This will pull the modulating sine wave out of the exponent, allowing for easier manipulation.

$$10^{x}=\sum_{n=0}^{\infty }\frac{x^{n}\;{\text{ln}}^{\left(n\right)}(10)}{n!},$$ (5)

where ln (x) is the natural logarithm or $\;{\text{log}}_{e}(x)$. By substituting this into the equation for the modulator, Eq. (4) becomes

$$M_{\;\text{Exp}\;}(f_{n},t)=\sum_{a=0}^{\infty }\frac{1}{a!}{\left(\frac{a\;\;\text{ln}\;(10)}{20}\right)}^{a}\;{\text{sin}}^{a}(2\;\pi \;\omega \;t+\Phi_{n}).$$ (6)

Several expressions make repeated reappearances, therefore, it is prudent to make substitutions for clarity and brevity. The argument to the sine function in the modulator is substituted with $\Theta_{n}(t)\equiv 2\;\pi \;\omega \;t+\Phi_{n}$, and a constant factor appearing as the amplitude is represented with C≡m  ln (10)/20. Using these simplifying expressions, the modulator becomes

$$M_{\;\text{Exp}\;}(f_{n},t)=\sum_{a=0}^{\infty }\frac{C^{a}}{a!}\;{\text{sin}}^{a}(\Theta_{n}(t)).$$ (7)

To simply this, an explicit, analytic substitution for $\;{\text{sin}}^{n}(\theta )$, called the sine power-reduction formula^14^ is used

$$\;{\text{sin}}^{n}(\theta )=\{\begin{array}{ll} \frac{2}{2^{n}}\sum_{k=0}^{(n-1)/2}{\left(-1\right)}^{(n-1)/2+k}\left(\begin{array}{c} n\\ k \end{array}\right)\text{sin}\;\left((n-2k)\;\theta \right), & n\;\text{is}\;\text{odd},\\ \frac{1}{2^{n}}\left(\begin{array}{c} n\\ \frac{n}{2} \end{array}\right)+\frac{2}{2^{n}}\sum_{k=0}^{n/2-1}{\left(-1\right)}^{n/2+k}\left(\begin{array}{c} n\\ k \end{array}\right)\text{cos}\;\left((n-2k)\;\theta \right), & n\;\text{is}\;\text{even}, \end{array}$$ (8)

where the binomial coefficient is defined as

$$\left(\begin{array}{c} a\\ b \end{array}\right)\equiv \frac{a!}{(a-b)!\;b!}.$$ (9)

Substituting the sine power-reduction formula into the expression for the modulator $M_{\;\text{Exp}\;}(f_{n},t)$ [Eq. (6)] yields

$$M_{\;\text{Exp}\;}(f_{n},t)=\left\{\begin{array}{cc} \;\text{even} & \sum_{a=0}^{\infty }\frac{C^{a}}{2^{a}\;a!}\left(\begin{array}{c} \;a\;\\ \frac{a}{2} \end{array}\right)+\\ \;\text{even} & \sum_{a=2}^{\infty }\frac{C^{a}}{2^{a-1}\;a!}{\left(-1\right)}^{a/2}\sum_{k=0}^{a/2-1}{\left(-1\right)}^{k}\left(\begin{array}{c} a\\ k \end{array}\right)\text{cos}\;\left(\left(a-2k)\;\Theta_{n}(t\right)\right)+\\ \;\text{odd} & \sum_{a=1}^{\infty }\frac{C^{a}}{2^{a-1}\;a!}{\left(-1\right)}^{(a-1)/2}\sum_{k=0}^{(a-1)/2}{\left(-1\right)}^{k}\left(\begin{array}{c} a\\ k \end{array}\right)\text{sin}\;\left(\left(a-2k)\;\Theta_{n}(t\right)\right) \end{array}\right\},$$ (10)

where *even* and *odd* refer to only summing over the even and odd terms, respectively.

Separating the unitary, cosine, and sine terms into separate expressions for convenience and collecting the similar sine and cosine terms, the modulation terms become

$$\sum_{a=0}^{\infty }\frac{C^{2a}}{2^{2a}\;a!\;a!},$$ (11a)

$$\text{even}\;\;\sum_{a=2}^{\infty }\frac{2C^{a}}{2^{a}}{\left(-1\right)}^{a/2}\;\text{cos}\;\left(a\Theta_{n}(t)\right)\sum_{k=0}^{\infty }\frac{C^{2k}}{k!(k+a)!2^{2k}},$$ (11b)

$$\text{odd}\;\;\sum_{a=1}^{\infty }\frac{2C^{a}}{2^{a}}{\left(-1\right)}^{(a-1)/2}\;\text{sin}\;\left(a\Theta_{n}(t)\right)\sum_{k=0}^{\infty }\frac{C^{2k}}{k!(k+a)!2^{2k}}.$$ (11c)

Next, the *modified Bessel function of the first kind* is required,

$$I_{\nu }(z)\equiv {\left(\frac{z}{2}\right)}^{\nu }\sum_{k=0}^{\infty }\frac{z^{2k}}{2^{2k}\;k!\;\Gamma (\nu +k+1)},$$ (12)

where the Gamma function, when the argument is restricted to natural numbers, is equal to a factorial

Γ(n)=(n−1)! ∀ n∈ℕ. (13)

When values of *ν* are constrained to the set of natural numbers (as will be true in this case), Eq. (12) simplifies to

$$I_{\nu }(z)={\left(\frac{z}{2}\right)}^{\nu }\sum_{k=0}^{\infty }\frac{z^{2k}}{2^{2k}\;k!\;(\nu +k)!}.$$ (14)

Expressions following this form appear in Eqs. (11a), (11b), and (11c). Using the definition of the *modified Bessel function of the first kind* and substituting these expressions back into Eq. (10) yields

$$M_{\;\text{Exp}\;}(f_{n},t)=\left\{\begin{array}{cc} I_{0}\left(C\right)+ & \\ \text{even} & 2\sum_{k=2}^{\infty }{\left(-1\right)}^{k/2}I_{k}\left(C\right)\text{cos}\;\left(k\;\Theta_{n}(t)\right)+\\ \text{odd} & 2\sum_{k=1}^{\infty }{\left(-1\right)}^{(k-1)/2}I_{k}\left(C\right)\text{sin}\;\left(k\;\Theta_{n}(t)\right) \end{array}\right\}.$$ (15)

Substituting this back into the generalized form of modulated noise [Eq. (1)] and multiplying out yields

$$S(t)=\sum_{n=1}^{N}A_{n}\left\{\begin{array}{cc} & I_{0}\left(C\right)\text{sin}\;(2\pi f_{n}t+\phi_{n})+\\ \text{even} & 2\sum_{k=2}^{\infty }{\left(-1\right)}^{k/2}I_{k}\left(C\right)\text{cos}\;\left(k\;\Theta_{n}(t)\right)\text{sin}\;\left(2\pi f_{n}t+\phi_{n}\right)+\\ \text{odd} & 2\sum_{k=1}^{\infty }{\left(-1\right)}^{(k-1)/2}I_{k}\left(C\right)\text{sin}\;\left(k\;\Theta_{n}(t)\right)\text{sin}\;\left(2\pi f_{n}t+\phi_{n}\right) \end{array}\right\}.$$ (16)

Simplifying Eq. (16) with the trigonometric product rules yields the following expression:

$$S(t)=\sum_{n=1}^{N}A_{n}\left\{\begin{array}{cc} & I_{0}\left(C\right)\text{sin}\;\left(2\pi f_{n}t+\phi_{n}\right)+\\ \text{even} & \sum_{k=2}^{\infty }{\left(-1\right)}^{k/2}I_{k}\left(C\right)\text{sin}\;\left(2\pi (f_{n}+k\;\omega )\;t+\phi_{n}+k\;\Phi_{n}\right)+\\ \text{even} & \sum_{k=2}^{\infty }{\left(-1\right)}^{k/2}I_{k}\left(C\right)\text{sin}\;\left(2\pi (f_{n}-k\;\omega )\;t+\phi_{n}-k\;\Phi_{n}\right)+\\ \text{odd} & \sum_{k=1}^{\infty }{\left(-1\right)}^{(k+1)/2}I_{k}\left(C\right)\text{cos}\;\left(2\pi (f_{n}+k\;\omega )\;t+\phi_{n}+k\;\Phi_{n}\right)+\\ \text{odd} & \sum_{k=1}^{\infty }{\left(-1\right)}^{(k-1)/2}I_{k}\left(C\right)\text{cos}\;\left(2\pi (f_{n}-k\;\omega )\;t+\phi_{n}-k\;\Phi_{n}\right) \end{array}\right\}.$$ (17)

As a useful comparison, the linear modulation of Eq. (2) can be expanded to take the form

$$S(t)=\sum_{n=1}^{N}A_{n}\left\{\begin{array}{c} \text{sin}\;(2\pi f_{n}t+\phi_{n})\\ \;+\frac{m}{2}\text{cos}\;(2\pi (f_{n}-\omega )\;t+\phi_{n}-\Phi_{n})\\ \;-\frac{m}{2}\text{cos}\;(2\pi (f_{n}+\omega )\;t+\phi_{n}+\Phi_{n}) \end{array}\right\}.$$ (18)

A common interpretation of the linear modulation result above is that each carrier tone becomes the sum of three waves. The base wave is of amplitude *A_n_*, frequency *f_n_*, and phase $\phi_{n}$, and the remaining two are sidebands with frequencies of $f_{n}+\omega$ and $f_{n}-\omega$, phases of $\phi_{n}+\Phi_{n}+\pi$ and $\phi_{n}-\Phi_{n}$, and both with amplitudes of $\frac{1}{2}A_{n}m$ (see Table I).

In this context, Eq. (17) can be interpreted as a base tone plus an infinite number of diminishing pure-tone sidebands (see Table II). This representation is convenient because the sidebands converge to zero fairly quickly, and a number of efficient numerical recipes for evaluating the modified Bessel function exist,^13^ and it is included in standard matlab functions like besseli.

Representing STM in this way enables quickly and efficiently composing a stimulus in the frequency domain from many thousands of carrier tones and generating the time-domain representation with a single inverse discrete Fourier transform (IDFT). This method is easily fast enough to be used for real-time stimulus generation—decreasing the computation time for a 1-s stimulus by over 3 orders of magnitude to less than 40 ms (see Sec. IV B).

## IV. STRENGTHS AND LIMITATIONS

Whereas the solution derived for representing exponential modulation is explicit and analytic, making use of it is not without necessarily invoking some simplifying assumptions.

### A. Error

**FIG. 1.**
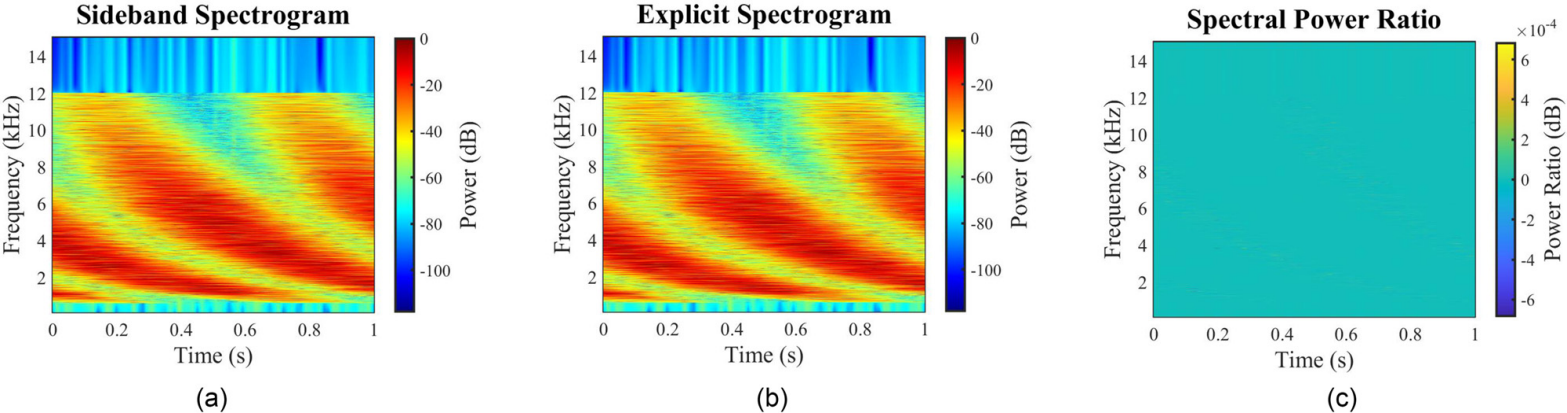
(Color online) Spectrographic analysis of sideband-based STM. Multiple comparisons of the proposed sideband-based generation method with a classic numerical solution for a midpoint-to-peak modulation depth of 20 dB (40 dB peak-to-valley). (a) Spectrogram of the STM created with the proposed Bessel function sideband approach using a sideband extent of five (ten sidebands), (b) spectrogram of the STM created by exhaustively evaluating the explicit form for exponential modulation, and (c) ratio of the Bessel function sideband-generated stimulus to the explicit form of the stimulus. Note that the colormap limits are $\pm 6\times 10^{-4}$ dB for the spectral power ratio.

Although the analytic representation of exponential modulation is expressed as a sum over an infinite number of sidebands, in practice, this expression does not require the inclusion of many terms to be highly accurate. Figure 1 demonstrates a direct comparison between the proposed sideband approach [Fig. 1(a)] and the explicit evaluation of the modulation [Fig. 1(b)]. One stimulus exemplar was generated with each method, using the same carrier component frequencies, amplitudes, phases phases, and 20-dB midpoint-to-peak modulation. The differences between the respective spectrograms, which are equivalent to the ratios in the spectral power density, visualzied in Fig. 1(c) show the signals to be nearly identical.

**FIG. 2.**
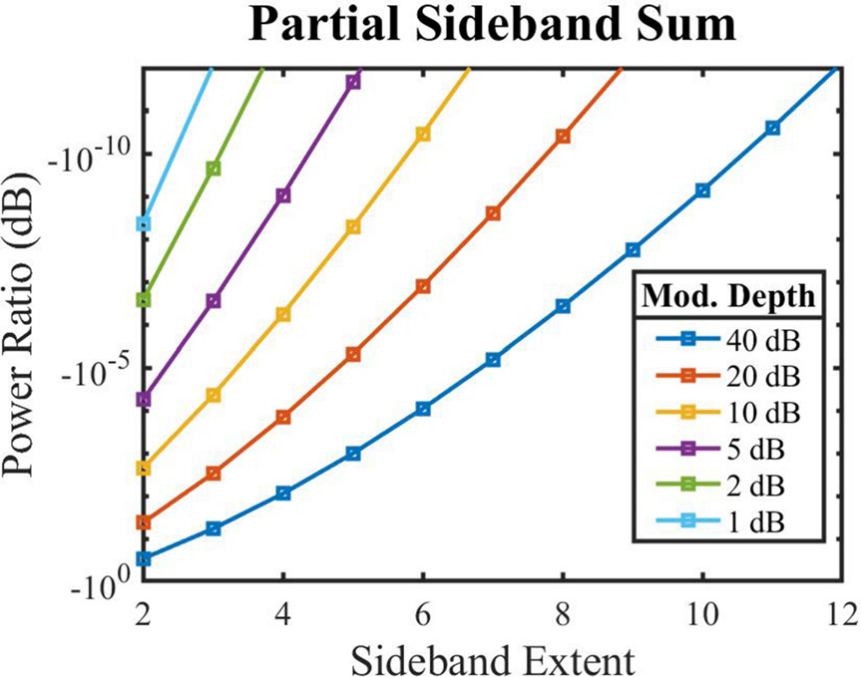
(Color online) The ratio between the energy of each term in the partial sum over a limited number of sidebands and the energy of the complete infinite sum, expressed in decibels and shown for several midpoint-to-peak modulation depths. Because sidebands are distributed symmetrically about the carrier tone, they are counted in terms of “sideband extent,” which is half of the total number of sidebands. The visualized “power ratio” value can be interpreted as the energetic contribution of the omitted terms relative to the entire sum. The values converge to zero quickly enough such that few total datapoints are visible when visualized with a linear ordinate axis.

Figure 2 visualizes how quickly the sum over the sidebands converges to the proper infinite sum. It is worth noting that the sideband convergence ordinate is in units of decibels plotted on a logarithmic scale, and the linear features shown in this scale represent hyper-exponential convergence. Thus, the midpoint ordinate value of $-10^{-5}$ represents a -0.00001-dB difference between the partial sum and the complete sum, or effectively 0.00001 dB of “missing” energy. The trend is that the greater the depth of modulation, the more sidebands are required to capture the dynamics. Even an envelope with a 40-dB midpoint-to-peak modulation depth (80-dB peak-to-valley) requires only 21 terms (20 sidebands between −10*ω* and +10*ω* plus the fundamental frequency) to have a power spectrum accurate to one part in 10^9^ in decibels. The perceptual relevance of these small level differences is difficult to evaluate because of the many different stimulus types that can be created and the variety of perceptual tasks that one might use with such stimuli. Using the extremely conservative estimate that a total energy difference of 0.01 dB due to omitted sidebands should be physiologically undetectable, a sideband extent of four would be sufficient for modulation depths up to 40 dB. In comparison to perceptual data, the best thresholds obtained by incrementing a single component in a tonal complex correspond to about a −20-dB signal-to-standard ratio,^15^ which corresponds to a level difference of 0.828 dB.^16^

### B. Computational complexity

As previously alluded to, the DFT is a powerful tool. When feasible, generating stimuli in the frequency domain can be several orders of magnitude faster than comparable time-domain methods. For example, generating a 1-s sample of STM with 10 000 carrier tones in the frequency domain with the proposed algorithm is consistently over 1200 times faster than exhaustively evaluating the explicit form of the stimulus in the time domain.^17^ In fact, the difference in computational complexity between the linear and exponential modulation cases in the frequency domain is relatively small as the cost for stimuli of duration greater than even 100 ms is dominated by the cost of the DFT [a fast Fourier transform (FFT) in this case], which is represented equally in the complexity of both algorithms. In the aforementioned test case, it took on average 38 ms to generate 1 s of exponential STM composed of 10 000 carrier tones in the frequency domain, whereas 48.7 s were required to generate the same stimulus in the time domain.

This comparison can also be made in the language of algorithmic complexity. In terms of big O complexity, using *C* for the carrier tone count, *N* for the number of samples, and *B* for the number of sidebands used, the complexity of the explicit calculation is O(CN), whereas the sideband-based approach is O(N  ln  N) when *N* is a power of two and, thus, the FFT can be used. The latter would contain the addition of a term of *BC*, but it falls away due to insignificance. However, the algorithmic complexity only captures part of the computational savings of the frequency domain approach as a result of the unequal cost of evaluating different arithmetic operations. In particular, the exponentiation and trigonometric function evaluations, which are evaluated *B* times for every sample in the explicit form, are notably slower than the addition and multiplication operations that form the backbone of the FFT.

Consideration of computational efficiency is particularly pertinent in light of Resnick *et al.*,^10^ demonstrating the effects of the spectral aliasing that occurs when generating STM with an insufficient carrier density. Low carrier densities have been used in the past to offset computational constraints, but the proposed sideband approach renders such carrier degradation optional.

## V. VALIDATION

Validation of the proposed sideband exponential stimulus generation technique can be further explored using metrics of the spectro-temporal envelope fluctuations as the basis for comparison between the explicit calculations and a third existing exponential technique as described by Chi *et al.*^1,18^ Direct, meaningful evaluation of the envelope of STM with its characteristic spectral *and* temporal variation is challenging. However, both pure SM and pure TM allow for much simpler approaches in accessing and analyzing the envelope. The modulation envelope in SM is directly inscribed into the spectrum just as the modulation envelope in TM appears in the time-domain envelope. Because all of the approaches analyzed here are composed of a sum over modulated carrier tones, the difference between SM and STM is the omission of the TM term in Eq. (4), *ω*, which advances the envelope phase with time, whereas the Φ(f) term that advances the envelope phase with frequency in the same equation is omitted in the case of TM. The envelope of the spectrum of pure SM is the focus of this analysis to best resolve any frequency-sensitive artifacts that might be present in the tested generation methods.

To evaluate the spectral envelope, 100 stimulus exemplars were computed with each method at each of 20 selected modulation depths, using maximum-density carriers with amplitudes sampled from a Rayleigh distribution and scaled to match a bandpass filter (−32 dB/octave) from 400 to 3200 Hz. The SM frequency was 2 cycles/octave, and the starting phase of the modulator was randomized. For each exemplar, two metrics of the spectral-envelope fluctuation were computed.

**FIG. 3.**
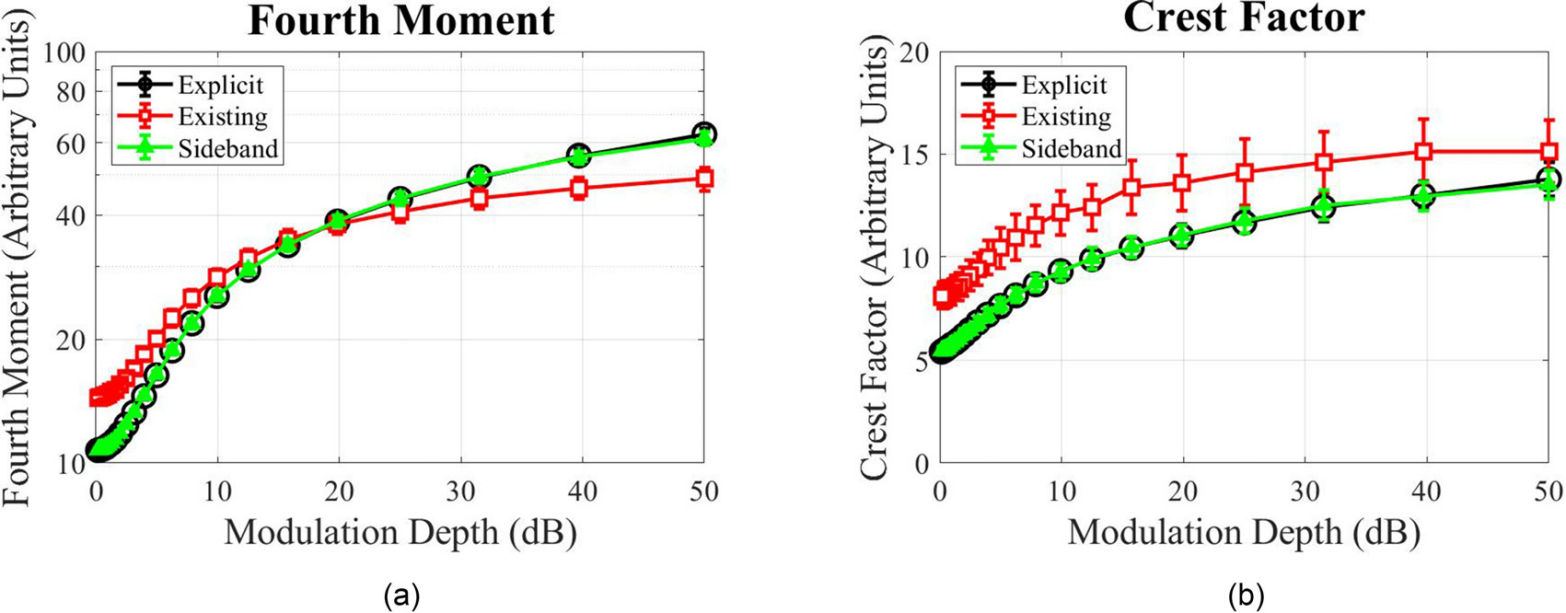
(Color online) Metrics of spectral envelope fluctuations in SM compared among three generation methods: black open circles for the explicit evaluation, red open squares for the existing method, and green filled triangles for the proposed sideband method. The calculations were performed on 100 exemplars across each of 20 modulation depths for each stimulus generation method. (a) The fourth moment of the spectrum (ordinate) as a function of the SM depth (abscissa). Error bars indicate the standard deviation across the 100 exemplars. (b) The crest factor of the spectral envelope (ordinate) as a function of the SM depth (abscissa). Error bars indicate the standard deviation across 100 samples.

The first metric used to analyze the signals was the normalized fourth moment (*M*_4_) of the spectrum,^19,20^

$$M_{4}=\frac{{\bar{E}}^{\;4}}{{\left(\bar{E^{2}}\right)}^{2}},$$ (19)

where *E* is the stimulus spectrum, obtained from a DFT. The fourth moment characterizes the degree of fluctuations of a signal and could potentially capture issues like inadequate curvature or frequency-dependent modulation artifacts. Figure 3(a) shows the normalized fourth moment of the spectrum as a function of modulation depth (peak-to-valley difference in dB) from 0 to 50 dB. The three functions represent the explicit evaluation (black, open circles), the existing method (red, open squares),^1^ and the proposed sideband method (green, filled triangles). The most important observation in this context is that the proposed sideband method (green triangles) maps directly onto the explicit method (black circles), indicating that the minimal computational error discussed above has minimal effect on modulation envelope curvature and depth. Also of potential interest is the fact that the normalized fourth moment values for the existing method (red squares) are quite different from the other two methods. For modulation depths below about 20 dB, the normalized fourth moment is greater than the other two methods, and for greater modulation depths, the normalized fourth moment is less.

The second metric was the crest factor, CF, of the spectrum,

$$\text{CF}=\frac{\left|E_{\text{peak}}\right|}{E_{\text{rms}}},$$ (20)

where $E_{\text{peak}}$ is the peak in the spectral envelope, and $E_{\text{rms}}$ is the RMS magnitude of the spectrum. The crest factor characterizes the extrema of a signal and would reveal if a method suppressed or exaggerated transients and peaks. This metric, shown in Fig. 3(b), also confirms that the explicit method and proposed sideband method are nearly identical in terms of envelope peaks, whereas the existing method had an elevated crest factor (by about 36% on average across modulation depths).

Overall, these methods of quantifying spectral envelope fluctuations show that the explicit and proposed sideband methods yield roughly the same spectral envelope depth and both differ from the envelope of the existing method even when the same spectral envelope parameters are specified during stimulus generation. Although the differences between theses methods shown in Fig. 3 do point to potential envelope cues introduced by using the existing method, it is currently unknown what impacts, if any, the differences in envelope actually have on perception. With the proposed, more efficient method in hand, it will now be much easier to systematically examine exactly which spectral and temporal cues are responsible for sensitivity to STM and how this interacts with the stimuli that have been used to measure sensitivity in the past.

## VI. SUMMARY AND CONCLUSIONS

The derivation of an explicit, analytic expression for the sidebands necessary to capture exponential modulation has been presented. Where existing solutions capable of real-time execution either estimate this effect through coarser Fourier transforms or approximate filtering, the proposed solution solves for the precise sideband values necessary to capture the behavior. A set of metrics evaluating the spectral envelope demonstrate that the modulation generated by the proposed method more closely matches the expected form than does an accepted alternative. The degree to which the modulation envelope shape interacts with the auditory system and impacts STM detection is an area of study that is still evolving, and empirical evidence on the impact of differences in shape is limited. For example, Shamma and Versnel^21^ reported that modulation shape did not lead to a difference in ferret cortical single-unit responses. Isarangura *et al.*,^22^ however, reported that SM detection thresholds differed significantly based on the spectral envelope shape. Consequently, efficient methods of generation like the proposed sideband approach will help keep differences and potential errors in generation from slowing progress in our further understanding of the perceptual and physiological bases and implications of modulation sensitivity.

## APPENDIX A: PSEUDOCODE

In the interest of maximizing clarity and usability, a pseudocode making use of the sideband representation is presented below. For a given modulation depth *M*, carrier tone count *N*, sideband extent *B*, TM rate *ω*, modulator phases $\Phi_{n}$, carrier frequencies *f_n_*, carrier amplitudes *A_n_*, and carrier phases $\phi_{n}$.

Carrier tone frequencies, amplitudes, and phases would be as described in U Appendix B, $\Phi_{n}$ would follow the example in Eq. (3), and the sideband extent *B* need not be larger than ten for highly accurate values (see Fig. 2).

An example of a matlab code implementing this procedure and generating Fig. 1 is included as supplementary material.^23^

## APPENDIX B: CARRIER COMPOSITION

The frequencies and amplitudes used for carrier tones in the generation of STM critically determine spectral properties of the resulting stimulus like whether the underlying stimulus is white noise, pink noise, a tonal complex of a desired density (i.e., frequency spacing), or some other carrier entirely.

### 1. Linear frequency spacing

Mathematically, the simplest way to distribute carrier tones over a frequency range is to do so linearly. Noise generated this way, if no additional frequency-dependent factors are applied to the amplitude of the carrier tones, can have the long-term spectral shape and short-term statistical composition of white noise—a flat power distribution throughout the linear frequency space with Rayleigh (Chi square) distributed magnitudes. This means that, on average, the frequency ranges of 100–200 Hz and 1000–1100 Hz should contain the same power. For *N* carrier tones between frequencies $f_{\text{LB}}$ and $f_{\text{UB}}$,

$$\begin{array}{l} f_{n}=f_{\text{LB}}+(f_{\text{UB}}-f_{\text{LB}})\frac{n-1}{N-1},\\ A_{n}=R,\\ \phi_{n}=[0,2\pi ), \end{array}$$

where *R* represents random numbers sampled from a Rayleigh distribution, which can be approximated from a flat distribution as $R\approx \sqrt{-2\;\;\text{ln}\;([0,1])}$.

Pink noise has an even energy distribution throughout logarithmic frequency space. This means that, on average, the same power should be carried by components between 100 and 200 Hz as between 1000 and 2000 Hz, or doubling the frequency range of any spectral band should halve the power. If one wants to generate noise with a pink spectral quality with a linear frequency distribution, then an additional factor of $1/\sqrt{f_{n}}$ must be applied to the amplitude, giving

$$\begin{array}{l} f_{n}=f_{\text{LB}}+(f_{\text{UB}}-f_{\text{LB}})\frac{n-1}{N-1},\\ A_{n}=\frac{R}{\sqrt{f_{n}}},\\ \phi_{n}=[0,2\pi ). \end{array}$$

### 2. Exponential frequency spacing

An alternative method of handling the distribution of carrier tone frequencies is to do so exponentially. For sufficient densities, this is perceptually indistinguishable from the linear distribution,^11^ but using the methods described above requires much less computational effort be spent for broad stimuli. This is consistent with a fixed number of carrier tones per octave.

Generating noise using an exponential frequency distribution and no additional restrictions on the amplitude generates pink noise as it implicitly reduces the spectral power per octave by 3 dB. For *N* carrier tones between frequencies $f_{\text{LB}}$ and $f_{\text{UB}}$,

$$\begin{array}{l} f_{n}=f_{\text{LB}}{\left(\frac{f_{\text{UB}}}{f_{\text{LB}}}\right)}^{\left(n-1)/(N-1\right)},\\ A_{n}=R,\\ \phi_{n}=[0,2\pi ). \end{array}$$

Alternatively, to generate white noise from such a frequency distribution, one must recover the power lost to the frequency distribution by applying an additional factor to the amplitude. This leads to

$$\begin{array}{l} f_{n}=f_{\text{LB}}+(f_{\text{UB}}-f_{\text{LB}})\frac{n-1}{N-1},\\ A_{n}=R\sqrt{f_{n}},\\ \phi_{n}=[0,2\pi ). \end{array}$$
